# WITHDRAWN: Gender Dysphoria Preceding Intersex Recognition Demonstrates a Biophysiological Basis for Sex Identity

**DOI:** 10.21203/rs.3.rs-6623724/v2

**Published:** 2025-08-01

**Authors:** Alexander Stokes, Beverly Rice, Billy Wooton, Kartik Saini, Joshua Burkhart, Helen Turner

## Abstract

The full text of this preprint has been withdrawn by the authors as it was submitted and made public without the full consent of all the authors. Therefore, the authors do not wish this work to be cited as a reference. Questions should be directed to the corresponding author.

